# Outcome of patients with lung cancer treated with stereotactic body radiotherapy for bone oligometastases - a European multicenter cohort study

**DOI:** 10.1186/s13014-026-02855-4

**Published:** 2026-05-19

**Authors:** Sebastian Schäfer, Isabell Seiler, Panagiotis Balermpas, Camilla von Wachter, Mauro Loi, Daniela Greto, Anna Sabrina Schunn, Sophia Drabke, Kenneth Klischies, Olaf Wittenstein, Jochen Willner, Fabian Lohaus, Lena Kästner, Johannes Meents, Priska Bank, Marek Slavik, Petr Burkon, Richard Partl, Jörg Andreas Müller, Yvonne Dzierma, Thomas Mader, Maike Trommer, Eleni Gkika, Alexander Rühle, Matthias Guckenberger, Christos Moustakis, Thomas Brunner, Oliver Blanck, Judit Boda-Heggemann, Nils H. Nicolay, Franziska Nägler

**Affiliations:** 1 Department of Radiotherapy, University Hospital Leipzig, Stephanstraße 9a, Leipzig, Germany; 2 Comprehensive Cancer Center Central Germany, Partner Site Leipzig, Leipzig, Germany; 3 Department of Radiation Oncology, University Hospital Zurich, University of Zurich, Rämistr. 100, Zurich, CH-8091 Switzerland; 4 Radiation Oncology Unit, Azienda Ospedaliero-Universitaria Careggi, University of Florence, Florence, Italy; 5 Department of Radiation Oncology and Radiation Oncology, University Medical Center Mainz, Langenbeckstraße 1, 55131 Mainz, Germany; 6 Department of Radiation Oncology, University Medical Center Schleswig-Holstein, Kiel, Germany; 7 Department of Radiotherapy, Klinikum Bayreuth GmbH, Preuschwitzer Str. 101, 95445 Bayreuth, Germany; 8 Department of Radiotherapy and Radiation Oncology, Faculty of Medicine and University Hospital Carl Gustav Carus, TUD Dresden University of Technology, Fetscherstraße 74, Dresden, Germany; 9 National Center for Tumor Diseases (NCT), NCT/UCC Dresden, A Partnership Between DKFZ, Faculty of Medicine and University Hospital Carl Gustav Carus, TUD Dresden University of Technology, and Helmholtz-Zentrum Dresden- Rossendorf (HZDR), Dresden, Germany; 10 OncoRay – National Center for Radiation Research in Oncology, Faculty of Medicine and University Hospital Carl Gustav Carus, TUD Dresden University of Technology, Helmholtz-Zentrum Dresden-Rossendorf, Dresden, Germany; 11 German Cancer Consortium (DKTK), Partner Site Dresden, and German Cancer Research Center (DKFZ), Heidelberg, Germany; 12 Department of Radiation Oncology, University Medicine Mannheim, Medical Faculty Mannheim, Heidelberg University, Theodor-Kutzer-Ufer 1-3, Mannheim, Germany; 13 DKFZ Hector Cancer Institute at the University Medical Center Mannheim, Mannheim, Germany; 14 Clinic for Radiation Therapy and Radio-Oncology Gera, Waldklinikum Gera, Germany; 15 Department of Radiation Oncology, Masaryk Memorial Cancer Institute and Medical Faculty, Masaryk University, Brno, Czech Republic; 16 Department of Therapeutic Radiology and Oncology, Comprehensive Cancer Center, Medical University of Graz, Graz, 8036 Austria; 17 Austrian Cancer Center Network (ACCN), Vienna, Austria; 18 Department of Radiation Oncology, University Hospital Halle (Saale), Ernst-Grube-Str. 40, 06120 Halle (Saale), Germany; 19 Department of Radiation Oncology, Rostock University Medical Center, Rostock, Germany; 20 Department of Radiation Oncology, Kantonsspital Graubünden, Chur, Switzerland; 21 Department of Radiation Oncology, Faculty of Medicine and University Hospital Bonn, Bonn, Germany; 22 Center for Integrated Oncology CIO Aachen Bonn Cologne Duesseldorf, Department of Internal Medicine, Faculty of Medicine and University Hospital Cologne, Cologne, Germany

**Keywords:** Bone metastases, Oligometastasis, Lung cancer, Stereotactic radiotherapy, Stereotactic body radiotherapy, SBRT, Metastasis-directed therapy, MDT

## Abstract

**Introduction:**

Metastasis-directed radiotherapy is of increasing importance in the multidisciplinary management of oligometastatic non-small cell lung cancer (NSCLC), but outcome patterns for stereotactic body radiotherapy (SBRT) of bone oligometastases (BoM) remain insufficiently defined. We aimed to determine oncological outcomes and prognostic factors of SBRT for BoM of NSCLC.

**Materials and methods:**

Patients with NSCLC treated with SBRT for < 5 BoM between 2010 and 2024 at 15 European cancer centers were retrospectively analyzed. Outcomes included freedom from local recurrence (FFLR), progression-free survival (PFS), overall survival (OS), and adverse events.

**Results:**

With a median follow-up of 14 months (IQR: 7–24 months), 85 patients with 111 treated BoM were analyzed. The 2-year FFLR was 87.2% (95%-CI: 73.3%-94.1%). The 1-/2-year PFS for singular BoM was 60.1% (CI: 44.6%-72.5%)/ 39.9% (CI: 24.6%-54.8%), while for 2–3 BoM they amounted to 10.2% (95%-CI: 0.6%-35.8%) and 0%. In multivariable analysis, less favorable outcome for OS and PFS was associated with larger BoM (HR 1.003; *p* < 0.01 and HR 1.005, *p* < 0.001) and increased number of treated BoM (HR 1.72; *p* = 0.03 and HR 1.93; *p* < 0.01). Treatment was well tolerated, with fracture rates of 5.4% and no grade 4 and 5 adverse events.

**Conclusion:**

This multicenter cohort analysis revealed that SBRT of BoM from NSCLC appears to be an effective and well-tolerated treatment. Presence of singular BoM was a favorable prognostic factor. Prospective studies are needed to confirm these findings and to determine the role of SBRT in the multidisciplinary management of oligometastases.

**Supplementary Information:**

The online version contains supplementary material available at 10.1186/s13014-026-02855-4.

## Introduction

Lung cancer is among the most frequently diagnosed cancers globally [1]. With approximately 30–40% of metastases occurring in bones, the skeletal system is a key metastatic site, and survival with bone metastases appears to be worse than with central nervous system metastases [2–5]. The effectiveness of innovative systemic therapies, especially immune checkpoint inhibitors (ICI) in patients with bone metastases (BoM) from NSCLC is subject to controversy due to contradictory studies [6].

BoM are a significant cause of skeletal-related events (SREs), and approximately one-third of BoM occurs as complicated lesions, resulting in pain, pathological fractures, neurological impairments and hypercalcemia [7]. Palliative radiotherapy is a guideline-recommended treatment for BoM, providing pain relief and prevention of SREs, thereby maintaining quality of life with minimal side effects [8, 9]. In the management of oligometastatic lung cancer (OMLC), recent clinical trials have revealed the clinical benefit of stereotactic body radiotherapy (SBRT) as metastasis-directed therapy (MDT) [10–14]. SBRT appears to achieve superior and more durable pain relief compared to conventional radiotherapy [15]. In recent years, practice guidelines for SBRT of BoM have been published, intending an increase of standardization in these concepts [16–18]. The European Society for Radiotherapy and Oncology (ESTRO) practice guideline recommends a prescribed biologically effective dose (BED_10_) exceeding 50Gy for locally ablative SBRT of spine metastases, allowing different fractionation concepts using 1 to 5 fractions [16]. Nevertheless, uncertainties persist regarding the appropriate choice between locally ablative and palliative bone radiotherapy, as well as treatment details, such as optimum dose and treatment volume concepts [16, 19–22]. As stated in the interim analysis of the EORTC-ESTRO E2-RADIatE OligoCare study, commonly used SBRT concepts vary considerably in terms of dose and fractionation and consecutively also in terms of the BED [23]. Published approaches for SBRT of OMLC range from 1 fraction with 21–27 Gy, and 3 fractions with cumulative 25.5–33 Gy, to 5 fractions with up to 40Gy [12, 14].

As SBRT becomes more widely adopted in the multidisciplinary management of OMLC, it is important to ensure that practice patterns align with the best available evidence. Thus, the focus of our study was to evaluate routinely used bone SBRT concepts regarding oncological outcomes and safety, depending on the applied BED as well as, the number and localization of BoM.

## Materials and methods

### Patient cohort

We performed a retrospective, multicenter cohort study on SBRT of BoM within a project of the working group on radiosurgery and stereotactic radiotherapy of the German Society for Radiation Oncology (DEGRO). At 15 European cancer centers in Germany, Austria, Switzerland, the Czech Republic, and Italy, individual clinical and treatment data of patients receiving SBRT for BoM from NSCLC between 2012 and 2024, were collected and evaluated. BoM diagnosis was confirmed by imaging; histological confirmation of BoM was not mandatory. The classification of oligometastatic disease followed the established European consensus criteria [24]. Initial NSCLC treatment, application and type of concomitant and sequential systemic therapies during and/or within 4 weeks after SBRT of BoM, and presence of further metastases were recorded. SBRT parameters, including target volume definition, dose concepts, and plan parameters according to the International Commission on Radiation Units and Measurements (ICRU) report 91, were analyzed [25, 26]. BED was calculated with the formula BED = n×d×(1 + d/(α/β)) with n=number of fractions, d=dose per fraction, based on an α/β = 10Gy (BED_10_) [27]. A prescription BED_10_ of at least 40Gy for the target volume in a maximum of 10 fractions was requested as the minimal dose for bone SBRT, thereby excluding palliative concepts [28].

This study was approved by the leading Ethics Committee (127/24-ek) as well as by all local Ethics Committees of the participating centers in advance and followed the STROBE guidelines for reporting observational studies (Supplementary Data).

### Response assessment

The radiological findings of routinely administered post-SBRT imaging examinations (including CT, MRI, and PET scans) were retrospectively evaluated by specialists at the participating specialized centers, and then analyzed regarding local or distant tumor progression, and fractures following SBRT in the treated region. If patients received SBRT for more than one BoM, freedom from local recurrence (FFLR) analysis was performed independently for each lesion, while accounting for in-patient clustering. For calculation of FFLR following SBRT, local recurrence was determined as progression in imaging, and lesions without detection of recurrence were censored at the time of the most recent radiological findings. OS was calculated from the termination of SBRT until death from any cause. PFS was defined as the time interval from the end of SBRT until death or the occurrence of any imaging-confirmed progression of the tumor. Acute and chronic (≤/>90 days post SBRT) adverse events were documented according to Common Terminology Criteria for Adverse Events (CTCAE) version 5.0 [29].

### Statistical analysis

Statistical analyses were performed in Python (version 3.12.7) using custom-built scripts. Data preprocessing and management were conducted with pandas (v2.2.3) and NumPy (v2.2.1). Statistical computations and survival analyses were carried out using SciPy (v1.14.1) and the lifelines library (v0.30.0). Visualizations, including Kaplan–Meier curves, were generated with Matplotlib (v3.10.0). Patient and treatment characteristics were summarized using descriptive statistics, with medians reported together with the corresponding value range or 95% interval, as appropriate. Survival endpoints were evaluated using Kaplan–Meier estimators and uni- and multivariable Cox proportional hazards regression. Covariates entered into the regression models included Karnofsky performance scale (KPS in %), gross tumor volume (GTV in ml), BED_10_ GTV_mean_, synchronous versus metachronous occurrence, bone-only metastases, actionable targets, systemic therapy or target-specific treatment (concomitant or sequential to SBRT), and the total number of treated metastases. Age was excluded as it was not a significant factor in our preliminary studies [30]. Multivariable models were fitted on complete cases including only those variables with *p* < 0.10 in univariable testing. Due to the collinearity of the total number of treated metastases and solitary metastasis variables, only the former was included in the PFS multivariable model. Due to the small number of events, no multivariate analysis was performed for the FFLR. All model assumptions were assessed at a two-sided significance level of α = 0.05. Survival probabilities at 1 and 2 years were interpolated from the Kaplan–Meier estimates and reported with 95% confidence intervals (95%-CI). Group differences in survival distributions were compared using the log-rank test.

## Results

### Patient characteristics and treatment parameters

Of the original 149 bone metastases from NSCLC in our registry, we had to exclude 38 due to a fraction count > 10 or a BED_10_ <40Gy. Data of 85 patients with 111 BoM from OMLC treated with SBRT were analyzed, of whom 68/85 (80.0%) had a singular BoM, and 41/85 (48.2%) had a solitary BoM with no further metastases. Detailed patient characteristics are reported in Table 1.

**Table 1:** Patient characteristics

Variable	
*Patients (n=total)* *Number of treated BoM*: singular / multiple 2 BoM 3 BoM Treated BoM per patient: median (range) Localization spine / non-spine *Number of patients with only BoM* solitary / multiple *Further metastases known / unknown* Number of total metastases 1 / 2–5 / >5 Combined bone and visceral Lymph nodes Bone Visceral Cerebral Other	85 111 68 (80.0%) / 17 (20.0%) 9/85 (10.6%) 8/85 (9.4%) 1 (1–3) 54/111 (48.6%) / 57/111 (51.4%) 48/85 (56.5%) 41/85 (48.2%) / 7/85 (8.2%) 40/85 (47.1%) / 45/85 (52.9%) 41 (48.2%) / 34 (40.0%) / 10 (11.8%) 9/85 (10.6%) 8/85 (9.4%) 6/85 (7.1%) 5/85 (5.9%) 3/85 (3.5%) 9/85 (10.6%)
*Age -* Median in years (range)	65 (36–84)
*Karnofsky performance scale -* Median in % (95% range)	90% (70%-100%)
*Histology of primary NSCLC*	
Squamous cell carcinoma Adenocarcinoma Other NSCLC-histology No information available	9/85 (10.6%) 62/85 (72.9%) 5/85 (5.9%) 9/85 (10.6%)
*Molecular pathology of primary NSCLC (multiple possible)*	
PD-L1+ EGFR mutation KRAS mutation ALK translocation MET “Non-targetable” No information available	23/85 (27.1%) 10/85 (11.8%) 13/85 (15.3%) 3/85 (3.5%) 4/85 (4.7%) 10/85 (11.8%) 26/85 (30.6%)
*Initial therapy primary tumor* *(multiple treatment modalities possible)*	
Surgery Radiotherapy Systemic Therapy Other	23 (27.1%) 21 (24.7%) 31 (36.5%) 10 (11.8%)
*Occurrence of BoM*	
Time between initial diagnosis and BoM, median (range, months) Synchronous treatment Metachronous treatment Metachronous oligoprogression Metachronous oligorecurrence Repeat oligoprogression Repeat oligorecurrence Induced oligoprogression Induced oligorecurrence No information about type of metachronous disease	0.9 (0.0–57.0) 70/111 (63.1%) 41/111 (36.9%) 7/111 (6.3%) 11/111 (9.9%) 6/111 (5.4%) 9/111 (8.1%) 3/111 (2.7%) 2/111 (1.8%) 3/111 (2.7%)
*Characteristics of BoM*	
Radiological osteolytic Radiological osteoblastic Mixed osteolytic/ osteoblastic Soft tissue infiltration yes / no Spinal canal infiltration yes / no Symptomatic before SBRT yes / no	14/111 (12.6%) 54/111 (48.6%) 6/111 (5.4%) 19/111 (17.1%) / 67/111 (60.4%) 0/111 (0.0%) / 69/111 (62.2%) 43/111 (38.7%) / 52/111 (46.8%)
*Systemic therapy (multiple possible)*	
Cytotoxic chemotherapy concomitant / sequential Immunotherapy concomitant / sequential Targeted therapy concomitant / sequential No systemic therapy No information available	27/111 (24.3%) / 1/111 (0.9%) 28/111 (25.2%) / 1/111 (0.9%) 12/111 (10.8%) / 0/111 (0.0%) 53/111 (47.7%) 1/111 (0.9%)
*Imaging before treatment planning*: *(multiple imaging modalities possible)*	
Diagnostic CT MRI PET/CT Scintigraphy No information available	8 (7.2%) 42 (37.8%) 85 (76.6%) 2 (1.8%) 6 (5.4%)
*Treatment technique*	
LINAC (Photon FF / FFF) Robotic radiosurgery (CyberKnife)	40/111 (36.0%) / 56/111 (50.5%) 15/111 (13.5%)

Abbreviations: Bone metastases (BoM), PD-L1: Programmed death-ligand 1, EGFR: epidermal growth factor receptor, KRAS: Kirsten rat sarcoma viral oncogene mutation, ALK: anaplastic lymphoma kinase, CT: Computed Tomography, MRI: Magnetic Resonance Imaging, PET: Positron Emission Tomography, LINAC: Linear Accelerator, FF(F): Flattening-Filter(-Free), SBRT: Stereotactic Body Radiotherapy

Target volume definition was based on multimodal imaging such as PET-CT (85/111; 76.6%) and/or MRI (42/111; 37.8%) according to institutional standards and national recommendations [31, 32]. Considering target volumes, we observed mainly three different concepts as previously described [33, 34]. The predominantly used concept for non-spine lesions was the expanded GTV concept (GTV + safety margin) with 43/59 (72.9%) lesions, followed by a simultaneously integrated boost (SIB) used in 8/59 (13.6%) lesions. For spine localization, the SIB-concept with a high dose volume to the metastasis and an additional larger low-dose volume to the entire vertebra was used for 33/54 lesions (61.1%). The second most common concept was delineation with the accompanying spinal compartment for 12/54 (22.2%) metastases [35]. For 7/54 (13.0%) spine lesions, the expanded GTV concept was used.

For the entire cohort, median nominal prescribed dose (for SIB: prescribed dose to the high dose volume) was 35 Gy (range: 16.0–48.5 Gy) in 5 (range: 1–10) fractions (Table 2). Considering the prescribed dose concepts separately according to the localization of the treated BoM, it was found that 5 × 8 Gy (11/54; 20.4%), 5 × 7 Gy (10/54; 18.5%), and 10 × 4 Gy (10/54; 18.5%) were most commonly used for spine BoM. For non-spine BoM, 5 × 8 Gy (13/57; 22.8%) and 3 × 10Gy (12/57; 21.1%), followed by 5 × 7 Gy (11/57; 19.3%) were most frequently applied.

**Table 2:** Dose volume parameters according to ICRU91

	SIB concept	Expanded GTV concept	Compartment concept
Number of treatments	41 (36.9%)	48 (43.2%)	12 (10.8%)
Mean target volume (GTV) in cc (range)	11.8 (1.7–78.6)	6.0 (0.4–39.3)	30.0 (3.6–92.6)
Prescribed dose PTV, median in Gy (range)	40.0 (35.0–48.5)	35.0 (24.0–40.0)	30.0 (24.0–40.0)
Number of fractions, median (range)	10 (5–10)	5 (1–10)	3 (2–5)
Median PTV D_98%_ in Gy (range)	34.2 (22.5–45.0)	32.5 (21.2–40.7)	29.2 (22.6–39.7)
Median PTV D_50%_ in Gy (range)	41.4 (30.5–49.0)	35.4 (28.4–47.3)	34.2 (25.9–44.3)
Median PTV D_2%_ in Gy (range)	42.5 (36.2–51.7)	37.3 (30.8–50.5)	38.4 (28.0–49.1)
Median GTV_mean_ in Gy (range)	41.0 (35.0–48.5)	35.9 (29.6–49.2)	34.3 (25.8–45.4)
Median BED_10_ GTV_mean_ in Gy (range)	107.0 (79.7–144.3)	109.4 (94.0–169.2)	139.3 (86.4–152.9)
Median BED_10_ PTV D_2%_ in Gy (range)	71.8 (55.8–84.5)	65.3 (57.7–97.1)	76.9 (50.9–86.7)

Parameters presented according to the 3 treatment concepts

Abbreviations: SIB: Simultaneous Integrated Boost, GTV: Gross Tumor Volume, PTV: Planning Target Volume, BED: Biological Effective Dose with α/β = 10

In approximately half of treated BoM no systemic therapy was administered (53/111; 47.7%), and the majority of the other cases received some form of concomitant and/or sequential systemic therapy with one quarter cytotoxic chemotherapy (28/111; 25.2%) and another quarter immunotherapy (29/111; 26.1%, Table 1).

### Oncological outcome parameters

Median follow-up was 14 months (IQR: 7–24). During this period, 7 (6.2%) local progressions were documented, and 42 (49.4%) patients died.

Oncological outcome parameters were analyzed across subgroups defined by number (singular or 2–3), localization of treated BoM in spine or non-spine, and concomitant/ sequential systemic therapy.

FFLR at 1 and 2 years was 87.2% and 87.2% (95%-CI: 73.3%-94.1%) with a plateau appearing in the curve, respectively. In Kaplan-Meier-analysis, no dose dependency for FFLR was observed with regard to the applied BED_10_ of the SBRT concepts analyzed. Of the 7 local recurrences, 1 (14.3%) occurred in spine (compartment concept, 2 × 12 Gy), the other 6 (85.7%) in non-spine localizations (mainly in pelvis, all expanded GTV concept: 4 lesions with 5 × 7–8 Gy, 2 with 3 × 10Gy).

**Fig. 1:**
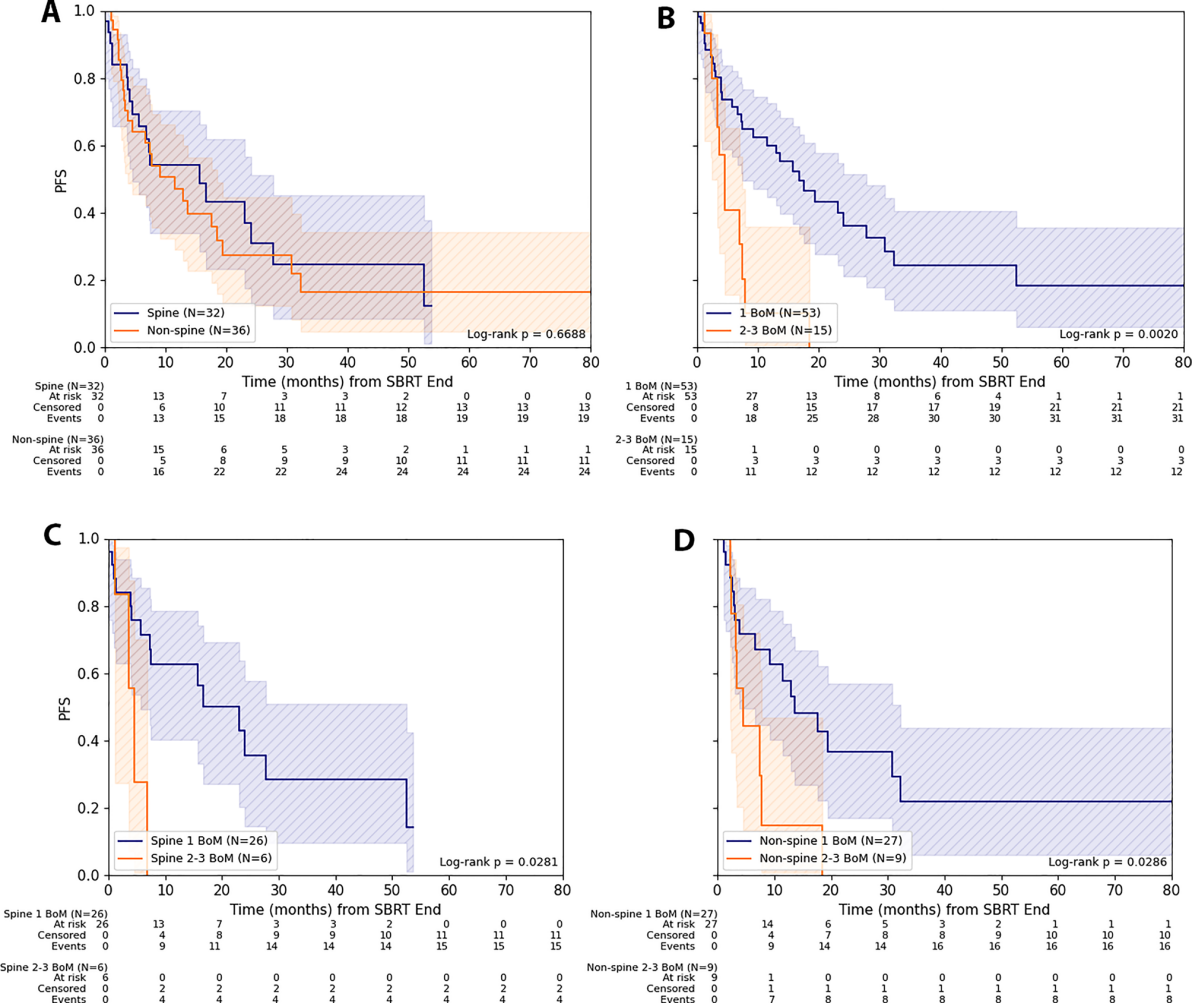
Progression-free survival for SBRT of bone metastasis from lung cancer. (**A**) Progression-free survival (PFS) depending on localization (spine/ non-spine) and (**B**) on number of treated bone metastases (BoM). (**C**) PFS depending on number of treated BoM in spine and (**D**) in non-spine

Patients with a singular treated BoM had a more favorable PFS than patients with 2–3 BoM (*p* = 0.002): The 1- and 2-year PFS for singular BoM ranged at 60.1% (CI: 44.6%-72.5%) and 39.9% (CI: 24.6%-54.8%), respectively, while for 2–3 BoM, 1-/2-year PFS amounted to 10.2% (CI: 0.6%-35.8%) and 0%, respectively. The more favorable PFS for singular BoM vs. 2–3 BoM was evident for both spine and non-spine localization (both *p* = 0.03). Considering only the localization of treated BoM in spine or non-spine without their number, the PFS did not differ significantly. Thus, 1-/2-year PFS for spine BoM was 54.1% (CI: 34.1%-70.4%) / 37.1% (CI: 17.7%-56.7%) and for non-spine localization 47.1% (CI: 29.0%-63.2%) / 27.3% (CI: 12.4%-44.6%), respectively (Fig. 1).

**Fig. 2:**
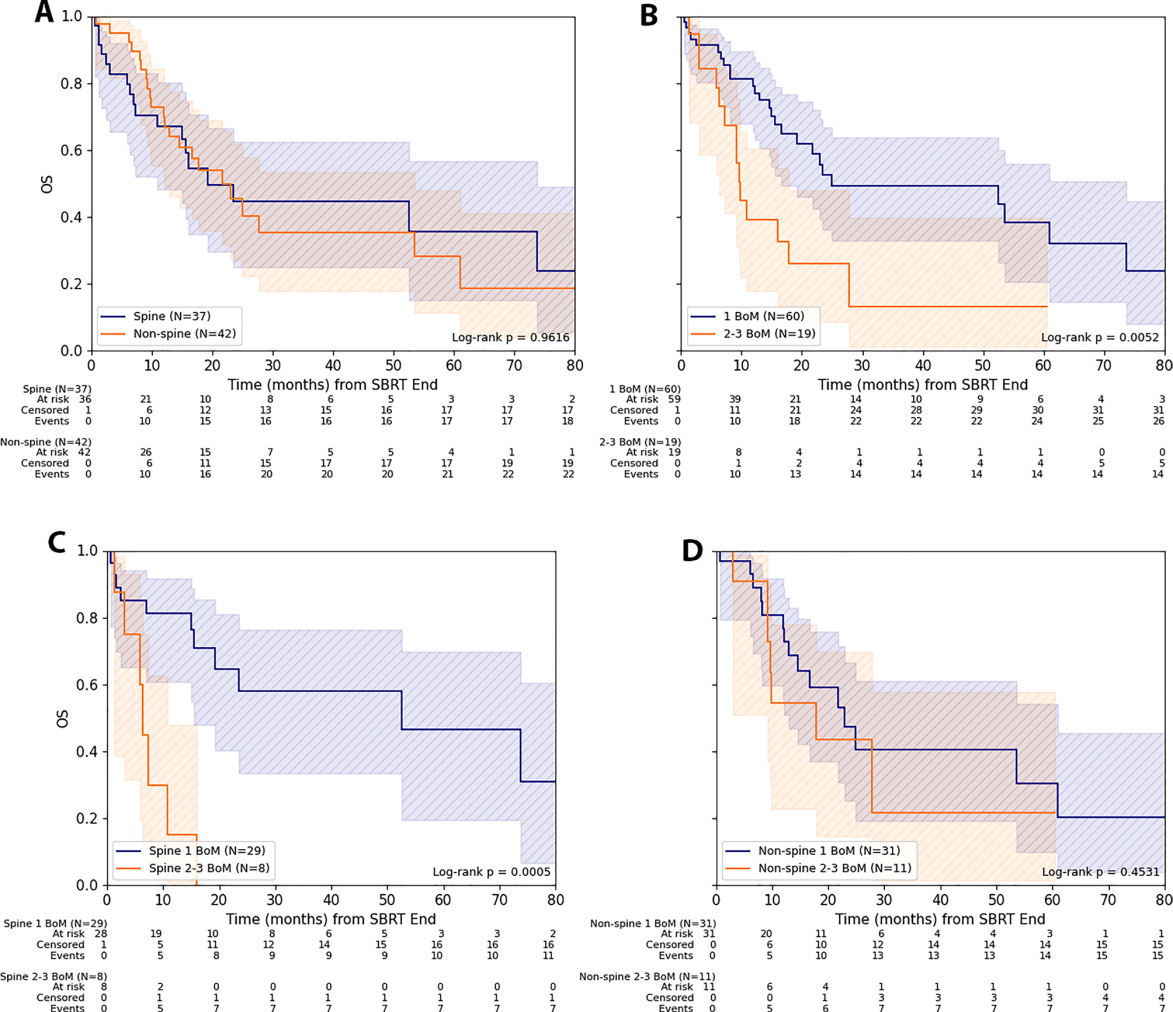
Overall survival for SBRT of bone metastasis from lung cancer. (**A**) Overall survival (OS) depending on localization (spine/ non-spine) for the entire cohort and (**B**) depending on number of treated bone metastases (BoM). (**C**) OS depending on number of treated BoM in spine and (**D**) in non-spine

For OS, there was a significant association between a singular treated BoM and improved OS, compared to the group with 2–3 treated BoM (*p* = 0.005). The results should be interpreted with caution, as the subgroup with 2–3 metastases was relatively small. The 1-/2-year OS for singular treated BoM in the entire cohort amounted to 79.2% (CI: 65.4%-87.9%) / 52.6% (CI: 36.1%-66.7%) versus 39.3% (CI: 17.8%-60.4%) / 26.2% (CI: 8.7%-48.0%) for 2–3 BoM. When considering the number of metastases in relation to their localization in the spine or non-spine, the described correlation was only significant for the subgroup with singular spine BoM (*p* < 0.001). No difference in OS could be observed regarding only localization of treated BoM: The 1-/2-year OS was 67.0% (CI: 48.1%-80.2%) / 44.6% (CI: 25.0%-62.5%) for spine BoM versus 69.8% (CI: 52.1%-82.1%) / 45.4% (CI: 27.1%-61.9%) for non-spine localization (Fig. 2).

Considering the additional administration of concomitant and/or sequential systemic therapy during SBRT, the combined treatment did not show any significant difference in outcome for either singular treated BoM or multiple treated BoM, whereby the number of patients in the respective groups was small (Supplementary Fig. 1). For this reason, it was not feasible to conduct a separate evaluation of patients based on the type of systemic therapy.

In univariable analysis, smaller GTV (in ml) was a favorable prognostic factor for both OS and PFS. A higher number of treated BoM was associated with worse PFS and OS (HR: 2.03; 95%-CI: 1.36–3.02; *p* < 0.001 and HR: 1.63; 95%-CI: 1.03–2.58; *p* = 0.04). Bone-only metastasis BoM was a favorable prognostic factor for PFS in univariable analysis (HR: 0.53; 95%-CI: 0.30–0.96; *p* = 0.04), while a higher number of treated BoM was associated with less favorable OS and PFS (HR: 1.63; 95%-CI: 1.03–2.58; *p* = 0.04, and HR: 2.03; 95%-CI: 1.36–3.02; *p* < 0.001). In multivariable analysis, favorable prognostic factors for OS were higher KPS (in %) and a smaller GTV (in ml). Additionally, for a higher number of treated BoM an association was observed with worse PFS and OS (HR: 1.93; 95%-CI: 1.21–3.08; *p* < 0.01 and HR: 1.72; 95%-CI: 1.06–2.81; *p* = 0.03). Details are presented in Table 3.

**Table 3:** Uni- and multivariable Cox proportional hazard regression analyses for overall survival and progression-free survival

Univariable	OS Events: 42		PFS Events: 50		FFLR Events: 7	
Variable	HR (95%CI)	*p*	HR (95%CI)	*p*	HR (95%CI)	*p*
KPS [%]	0.98 (0.95-1.00)	0.10	1.00 (0.97–1.03)	0.96	-	-
GTV [ml]	1.003 (1.001–1.004)	**< 0.01**	1.003 (1.001–1.005)	**< 0.01**	1.030 (1.014–1.045)	**< 0.001**
BED_10_ GTV_mean_	-	-	-	-	1.05 (0.97–1.14)	0.21
Synchronous / metachronous	1.09 (0.60–2.01)	0.77	0.84 (0.47–1.50)	0.54	-	-
Bone-only	0.82 (0.43–1.56)	0.54	0.53 (0.30–0.96)	**0.04**	-	-
Actionable targets	1.08 (0.58–2.01)	0.81	1.05 (0.59–1.87)	0.87	-	-
Systemic therapy (all)	0.88 (0.49–1.59)	0.67	0.84 (0.47–1.50)	0.55	-	-
Target-specific treatment	0.71 (0.39–1.30)	0.27	0.90 (0.51–1.62)	0.75	-	-
Number of treated BoM	1.63 (1.03–2.58)	**0.04**	2.03 (1.36–3.02)	**< 0.001**	-	-
**Multivariable**						
KPS [%]	0.97 (0.95-1.00)	0.07	-	-	-	-
GTV [ml]	1.003 (1.001–1.004)	**< 0.01**	1.005 (1.003–1.007)	**< 0.001**	-	-
Bone-only	-	-	0.58 (0.31–1.09)	0.09	-	-
Number of treated BoM	1.72 (1.06–2.81)	**0.03**	1.93 (1.21–3.08)	**< 0.01**	-	-

Abbreviations: OS: overall survival, PFS: progression free survival, FFLR: freedom from local recurrence, HR: hazard ratio, KPS: Karnofsky performance scale, GTV: Gross Tumor Volume, BED_10_ GTV_mean_: biologically effective dose (α/β = 10Gy), BoM: bone metastases. p-values < 0.05 are shown in bold

### SBRT-related adverse events

The rate of adverse events was low (24/111, 21.6%) with no grade-4 or 5 adverse events. Two acute grade-3 pain events were documented. Reported fracture rate related to SBRT was 2/111 (1.8%) for acute (localization: ribs) and 4/111 (3.6%) for delayed fractures (localization: 3 spine and 1 pelvis). Delayed fractures occurred 4.8-, 12.9-, 19.2-, and 26.3-months post SBRT (Table 4).

**Table 4:** SBRT-related acute and chronic adverse events, reported according to Common Terminology Criteria for Adverse Events (CTCAE) version 5.0

Acute side effects					
CTCAE Term	Grade 1 n (%)	Grade 2 n (%)	Grade 3 n (%)	Grade 4 n (%)	Grade 5 n (%)
Fatigue	5 (4.5%)	1 (0.9%)	-	-	-
Pain	2 (1.8%)	2 (1.8%)	3 (2.7%)	-	-
Diarrhea	2 (1.8%)	-	-	-	-
Radiodermatitis	1 (0.9%)	-	-	-	-
Nausea	1 (0.9%)	-	-	-	-
Paresthesia	1 (0.9%)	-	-	-	-
Fracture post SBRT	1 (0.9%)	1 (0.9%)	-	-	-

Chronic side effects					
	Grade 1 n (%)	Grade 2 n (%)	Grade 3 n (%)	Grade 4 n (%)	Grade 5 n (%)
Fracture post SBRT	2 (1.8%)	2 (1.8%)	-	-	-

n=number and relative values in percentage of total number of lesions *n* = 111 (100%)

Abbreviations: CTCAE: Common Terminology Criteria for Adverse Events

## Discussion

To the best of our knowledge, this is the first European multicenter cohort analysis evaluating the outcomes of SBRT for BoM from OMLC with a specific emphasis on the effectiveness and reliability of widely employed SBRT practice patterns. We found an association with improved OS and PFS for patients receiving SBRT to singular BoM compared to those patients with 2 to 3 treated BoM, while the presence of actionable targets and target-specific treatments appeared to be no significant factor for survival. The high efficacy of SBRT for BoM with 1-/2-year FFLR of > 85% was accompanied by a favorable safety profile. This was especially reflected in the low fracture rates.

In our retrospective cohort with mainly singular treated BoM of OMLC, no spinal canal infiltration, and half of the lesions without clinical symptoms before SBRT, we observed excellent local control rates at 1 and 2 years. The relatively short follow-up period and the 33 patients who died during the first 24 months allow only a limited assessment of the 2-year outcomes. In a meta-analysis of 18 studies for international stereotactic radiosurgery society (ISRS) clinical practice guidelines on SBRT for non-spine BoM, pooled 2-year control was similar with 94%. In this meta-analysis, univariable analysis identified certain histologies such as lung, renal cell carcinoma and melanoma as predictors of local failure. Similar to our results, BED was not a significant predictor of local control [18]. However, it is not possible to determine with certainty, whether the absence of a significant effect of the BED on local control in our data actually reflects the biological equivalence of the concepts used, or whether this is biased by the different target volume concepts.

Additionally, it should be noted, that with these excellent control rates and low fracture rates, hypofractionated SBRT appears to effectively reduce serious SREs and thus maintain patients’ quality of life. A recently published multicenter randomized phase II clinical trial with prophylactic radiotherapy for high-risk, asymptomatic BoM with a quarter primary lung cancer patients, could demonstrate significant fewer hospitalizations due to SRE and even a significant survival benefit. Thereby, in the experimental radiotherapy arm of the study, more than one-third of patients with > 1 metastasis were enrolled [20].

Considering PFS and OS in our cohort, we observed a positive association for patients with only singular treated BoM and thus lower tumor burden, compared to patients with two to three BoM. This effect has been described in the past for solitary brain metastases, but was now also clearly visible in our retrospective study on SBRT of BoM from NSCLC [36]. These findings suggest that future prospective studies for SBRT should also consider stratification based on the number of metastases and not solely the criterion of oligometastatic disease, in order to identify subgroups that could benefit most from additional MDT, such as SBRT, in combination with standard of care systemic therapies. However, several clinical trials for MDT of oligometastatic disease, including mainly phase II and one phase III study, have revealed benefits for PFS and even for OS [10–14]. Nonetheless, it should be noted that, to date, there is a particular lack of data on SBRT in combination with or in comparison to the rapidly evolving systemic, particularly targeted therapies. The NorthStar trial assessed the impact of additional local therapy (> 50% radiotherapy) to Osimertinib on PFS in patients with EGFR-mutated metastatic NSCLC and revealed a significant prolonged PFS in patients with combined therapy, even in polymetastatic disease [37]. Another randomized phase II study on EGFR-mutated metastatic NSCLC also demonstrated prolonged PFS and OS with the addition of SBRT [38]. In the randomized, controlled phase II CURB trial on SBRT plus standard of care for oligoprogressive metastatic NSCLC after at least first line systemic therapy, adding of SBRT resulted in a more than four-times improvement in PFS. In this study 51/59 enrolled NSCLC patients had no targetable driver mutation, while in our cohort nearly half of the lesions had an actionable target [39].

Literature on ICI for BoM of NSCLC is controversial, with some studies suggesting that ICI alone is less effective in NSCLC with BoM, and others, not confirming reduction in effectiveness, in particular for combination of chemo-immunotherapies in patients with BoM [3–6, 40]. Interestingly, nearly half of our cohort was reported to have received no concomitant or sequential systemic therapy. This may have been due to the high proportion of singular BoM and also solitary metastasis in total. About one-quarter received chemotherapy and approximately another third ICI/targeted therapies. We did not observe a significant positive association for the outcome through the concomitant use of systemic therapies and SBRT of BoM, neither for the treatment of singular BoM, nor for 2 to 3 BoM. However, the combination of systemic therapy and SBRT was also well tolerated and did not result in any SBRT-related serious side effects during the follow-up period.

In our cohort of SBRT for OMLC, we observed a low fracture rate of 5.4%, which was slightly higher than in our dataset for SBRT of bone oligometastasis of prostate cancer [34]. This is remarkable, given that nearly half of the evaluated lesions were reported as osteoblastic lesions and are therefore considered to be less susceptible to fractures [41]. A large meta-analysis that combined data on spinal BoM from different primary cancers reported a total vertebral fracture rate of 9%, with 1.7% of these cases requiring surgical stabilization [42]. The comparatively low fracture rate in our cohort could be due to the increased use of hypofractionation and the relatively young patient age. A recent multivariable analysis described 1- and 2-year vertebral fracture rates of 8.4% and 12.3%, and revealed higher age, increased BED, and baseline fractures as risk factors associated with a higher risk of SBRT-related fractures [43].

The present study has several limitations, primarily due to its retrospective design and thus, the use of real-world data. Owing to the large number of participating centers, considerable heterogeneity was observed in terms of SBRT protocols, dose prescription, target or margin definition, treatment techniques, and follow-up procedures across institutions. Additionally, the reliance on radiological reports for the evaluation of local recurrence, in conjunction with the variability in imaging frequency and the assumed absence of standardized response assessment criteria in some cases, had the potential to exert an influence on the results of this study. The responsibility for assessing local recurrence or fractures following treatment belonged to the SBRT specialists at the participating centers. Thus, underreporting of local progression and fractures cannot be excluded. Another concern with the retrospective study design and the long evaluation period > 10 years are missing data, here, particularly with regard to driver mutations of the primary NSCLC. In order to attenuate these potential limitations and ensure a high degree of data homogeneity, this study exclusively focused on SBRT of bone oligometastasis of a specific subgroup characterized by NSCLC primary diagnosis, and a predefined prescribed BED_10_ in the target volume. Nevertheless, inconsistencies — particularly regarding driver mutation status — persist over the long treatment period, so that the positive influence of newer systemic therapies (especially ICIs) on outcomes may not have been adequately reflected.

In summary, our retrospective real-world cohort shows that SBRT of BoM from OMLC appears to be a well-tolerated and effective treatment. Prospective studies are needed to determine the role of MDT, such as SBRT, for oligometastatic NSCLC, especially in the light of evolving systemic therapies, and to clarify its effect on long-term clinical outcomes and quality of life.

## Supplementary Information

Below is the link to the electronic supplementary material.

Supplementary Material 1

Supplementary Material 2

## Data Availability

The dataset generated during the current study is available from the corresponding author on reasonable request.

## Abbreviations

BoM: Bone metastases
BED: Biologically effective dose
BED10: Biologically effective dose assuming α/β = 10Gy
CI: Confidence interval
CT: Computed tomography
CTCAE: Common Terminology Criteria for Adverse Events
DEGRO: German Society for Radiation Oncology
EGFR: Epidermal growth factor receptor
ESTRO: European Society for Radiotherapy and Oncology
EORTC: European Organisation for Research and Treatment of Cancer
FFLR: Freedom from local recurrence
GTV: Gross tumor volume
HR: Hazard ratio
ICRU: International Commission on Radiation Units and Measurements
ICI: Immune checkpoint inhibitors
ISRS: International Stereotactic Radiosurgery Society
KPS: Karnofsky performance scale
MDT: Metastasis-directed therapy
MRI: Magnetic resonance imaging
NSCLC: Non-small cell lung cancer
NumPy: Numerical Python
OMLC: Oligometastatic lung cancer
OS: Overall survival
Pandas: Python Data Analysis Library
PET: Positron emission tomography
PET-CT: Positron emission tomography–computed tomography
PFS: Progression-free survival
RT: Radiotherapy
SBRT: Stereotactic body radiotherapy
SIB: Simultaneously integrated boost
SREs: Skeletal-related events
STROBE: Strengthening the Reporting of Observational Studies in Epidemiology
