# Supplementary material for: Outcome of patients with lung cancer treated with stereotactic body radiotherapy for bone oligometastases - a European multicenter cohort study
Source: Radiat Oncol. 2026 May 19;21:77. doi: 10.1186/s13014-026-02855-4 (PMC13188523; doi:10.1186/s13014-026-02855-4)
Supplement: Supplementary file 1 — Supplementary Material 1 [file 13014_2026_2855_MOESM1_ESM.docx]

**Supplementary Data**

**Supplementary Figure 1: Outcome for SBRT of bone metastasis from lung cancer**

Progression-free survival (PFS) depending on concomitant/ sequential systemic therapy (Sys or no Sys) and number of treated bone metastases (BoM) with A) 2-3 treated BoM and B) singular treated BoM.

Overall survival (OS) depending on concomitant/ sequential systemic therapy and number of treated BoM with C) 2-3 treated BoM and D) singular treated BoM.

**B**

**A**


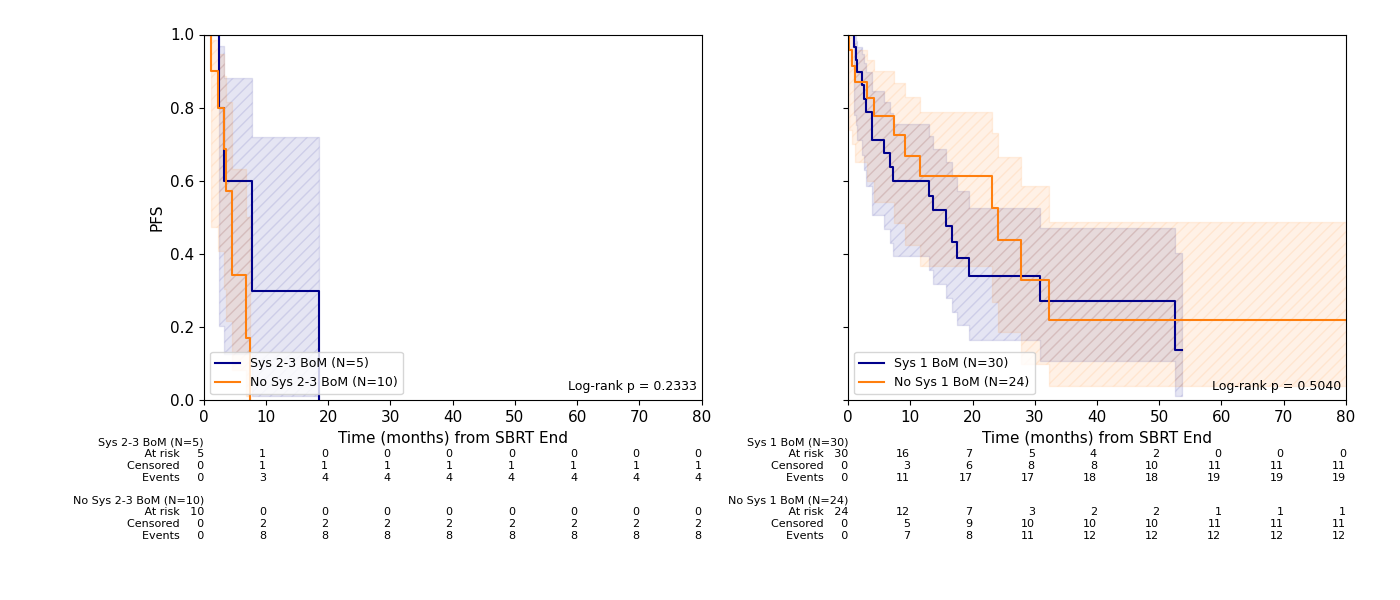


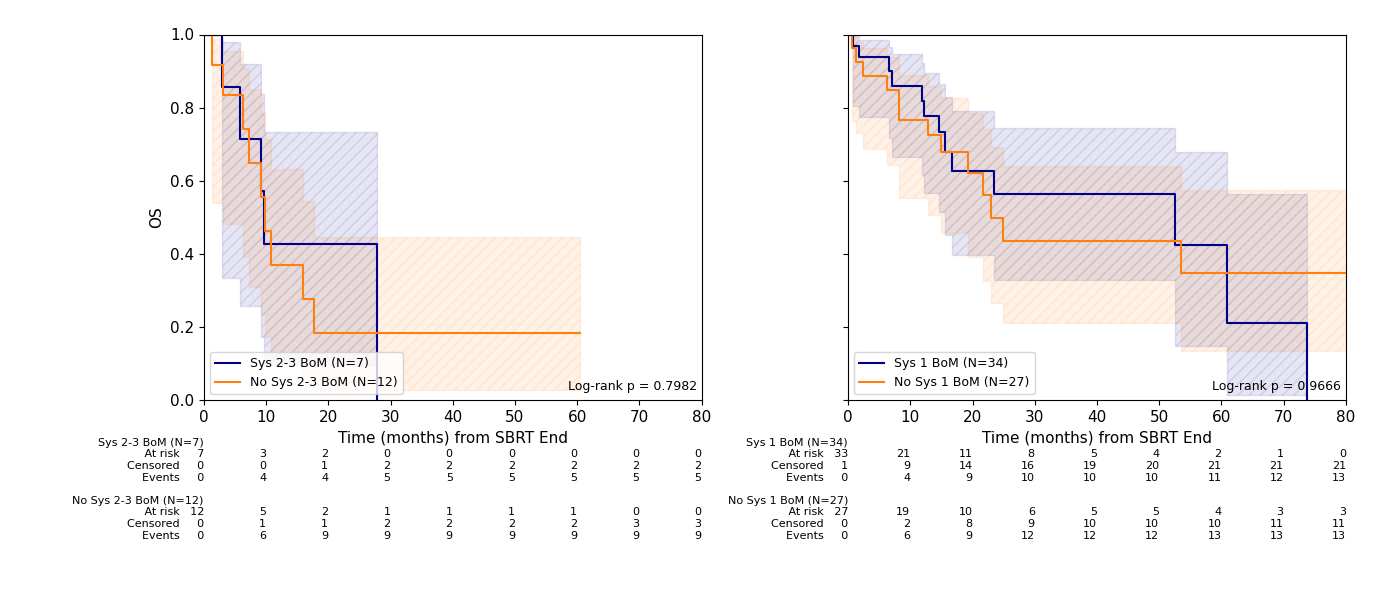


**C**

**D**
