# Supplementary material for: Outcome of patients with lung cancer treated with stereotactic body radiotherapy for bone oligometastases - a European multicenter cohort study
Source: Radiat Oncol. 2026 May 19;21:77. doi: 10.1186/s13014-026-02855-4 (PMC13188523; doi:10.1186/s13014-026-02855-4)

Supplementary Data

Supplementary Figure 1: Outcome for SBRT of bone metastasis from lung cancer
Progression-free survival (PFS) depending on concomitant/ sequential systemic therapy (Sys or no Sys) and number of treated bone metastases (BoM) with A) 2-3 treated BoM and B) singular treated BoM.
Overall survival (OS) depending on concomitant/ sequential systemic therapy and number of treated BoM with C) 2-3 treated BoM and D) singular treated BoM.

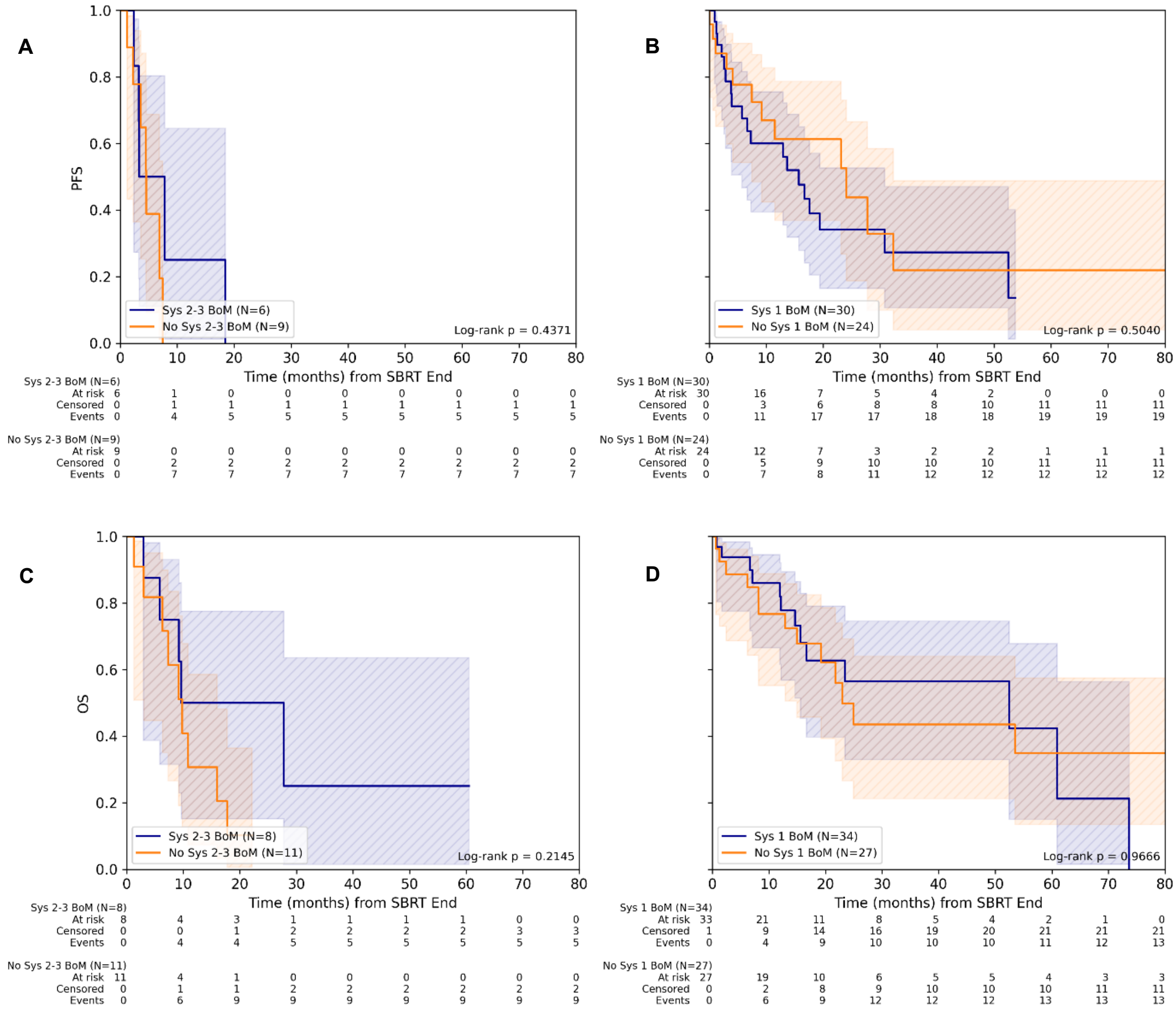

Supplement: Supplementary file 2 — Supplementary Material 2 [file 13014_2026_2855_MOESM2_ESM.pdf]
